# Effects of different types of non-cardiac surgical trauma on hippocampus-dependent memory and neuroinflammation

**DOI:** 10.3389/fnbeh.2022.950093

**Published:** 2022-08-10

**Authors:** Bo Lu, Hui Yuan, Lan Mo, Daofan Sun, Rongjun Liu, Han Zhou, Xiaojie Zhai, Ruichun Wang, Junping Chen, Bo Meng

**Affiliations:** ^1^Department of Anesthesiology, HwaMei Hospital, University of Chinese Academy of Sciences, Ningbo, China; ^2^Zhejiang Key Laboratory of Pathophysiology, School of Medicine, Ningbo University, Ningbo, China

**Keywords:** surgery, neuroinflammation, postoperative cognitive dysfunction, perioperative neurocognitive disorders, cognitive dysfunction

## Abstract

**Background:**

Older individuals have been reported to suffer from cognitive disorders after surgery. Various types of surgical trauma have been used to establish postoperative cognitive dysfunction (POCD) animal models in preclinical studies. However, few comparative analyses of these animal models were conducted.

**Methods:**

Tibial surgery, abdominal surgery, and extended abdominal surgery were performed on aged ICR mice to establish POCD models. Behavioral tests included open field, novel object recognition, fear conditioning, and Morris water maze tests. The Z-score methodology was adopted to obtain a comprehensive and integrated memory performance profile. The changes in hippocampal neuroinflammation were analyzed by ELISA, PCR, and immunofluorescence.

**Results:**

In this study, we found that each type of non-cardiac surgical trauma has a different effects on locomotor activity. Tibial and extended abdominal surgeries led to more significant cognitive impairment than abdominal surgery. Inflammatory cytokines peaked on postoperative day 1 and decreased to control levels on days 3 and 7. Hippocampal neuroinflammation indicators between the three surgery types on postoperative day 1 had no statistical differences.

**Conclusion:**

Overall, the type and intensity of non-cardiac surgical trauma can affect cognitive behavioral outcomes and central inflammation. The shortcomings and emerging issues of POCD animal research methods need to be further studied and solved.

## Introduction

The number of studies regarding learning, memory, and consciousness related to anesthesia continues to increase (Ghoneim and Block, 1997). For example, in preclinical studies, a group of anesthesiologists and psychologists are working to unravel the effects of different anesthetics on cognition or neurodevelopment (Le Freche et al., 2012; Zhang et al., 2015). In the past decade, an increasing number of researchers have been investigating the underlying mechanisms of cognitive dysfunction induced by the combination of anesthesia and surgery, known as postoperative cognitive dysfunction (POCD), which has been classified as perioperative neurocognitive disorders since Evered et al. (2018).

However, according to various clinical trials, anesthesia methods and anesthetics seem to have no effects on postoperative neurocognitive outcomes (Silbert et al., 2014). Preclinical studies using animal models have also confirmed that short-duration anesthesia does not affect cognitive function (Cibelli et al., 2010; Hovens et al., 2014; Lai et al., 2021). Hence, most researchers resort to different surgical trauma types to induce POCD in preclinical models, including open tibial fracture with intramedullary fixation, ischemia reperfusion of the upper mesenteric artery, exploratory laparotomy, right carotid artery exposure, partial hepatectomy, and splenectomy. Meanwhile, comparative analyses regarding these animal models are rarely performed. Recently, Hovens et al. (2016) compared the effects of trauma between cardiac and abdominal surgeries mainly based on the clinical background: more serious cognitive impairment after cardiac surgery versus non-cardiac surgery (Brown et al., 2018). In addition, cognitive impairment related to non-cardiac major surgery in the elderly is becoming a new research hot spot and was the focus of our current study.

According to the analyses regarding POCD animal models used in preclinical studies, we found that tibial fracture and abdominal surgeries were the most widely used (Supplementary Data Table 1). The tibial fracture surgery model was used for the first time for POCD by Cibelli et al. (2010). Referring to a fracture healing following surgery study Harry et al. (2008), Cibelli et al. (2010) developed a procedure consisting of two steps: open tibial fracture and intramedullary fixation for 10−20 min. Meanwhile, the abdominal surgery model could be traced back to Martin et al. (2005), in which the small intestine was exteriorized and vigorously manipulated with the thumb and forefinger. Then, some researchers modulated the method of laparoscopy to gently manipulate internal organs for 1 min using a sterile probe (Rosczyk et al., 2008). Furthermore, other researchers combined these two operations (Barrientos et al., 2012; Jia et al., 2015) into one that lasted 10−30 min, which was adopted by us in the present study. Here, we implemented a controlled study to explore the underlying mechanisms of POCD using the extended abdominal surgery model based on previous animal models, since POCD is more likely to happen after major surgeries.

The gap between POCD clinical and preclinical studies should be cautiously considered. Neuroinflammation hypothesis of POCD, for instance, originates and accumulates evidence mostly from preclinical studies. Thus, a consensus on POCD preclinical studies should be developed to reduce the gap. Using standardized animal models in preclinical studies of POCD is critical to ensuring consistency. Therefore, We aimed to investigate the effects of different types of surgical trauma on hippocampus-dependent memory and neuroinflammation in preclinical animal models, hoping to bridge the gap between POCD clinical and preclinical perspectives.

## Materials and methods

### Animals

Male ICR mice (12−14 months, 40−55 g) used in this study were purchased from the Experimental Animal Center of Zhejiang Province, China. All experimental procedures involving animals were approved by the Animal Care and Use Committee of Ningbo University in accordance with the guideline for the Care and Use of Laboratory Animals by the National Institutes of Health (NIH Publications No. 80-23). All animals were fed standard rodent diet and water *ad libitum* and were housed (four mice per cage) in a temperature-controlled animal facility with 12-h light/dark cycles.

### Surgery and anesthesia

Tibial surgery (TIS) consisted of an open tibial fracture of the left hind paw with intramedullary fixation in aseptic conditions as Terrando et al. (2010, 2011) conducted. The left hind paw was shaved, disinfected, and cut open to strip the periosteum, and osteotomy was performed. Then, a 0.38-mm pin was inserted into the intramedullary canal. After this fracture and fixation, the wound was irrigated with povidone-iodine and the skin sutured with 4/0 Prolene sutures.

Abdominal surgery (AS) was slightly modified and standardized by comprehensively referencing the procedures as previous (Barrientos et al., 2012; Xiao et al., 2018). Briefly, a 1.5-cm incision was made below the lower right rib. Then, a 0.5-cm sterile probe was inserted into the body cavity to manipulate the viscera and musculature for 1 min with a frequency of one movement per second. After that, 5-cm intestine was then exteriorized and manipulated between the surgeon’s thumb and forefinger for 1 min with one movement per second. Extended abdominal surgery (EAS) was performed by intensified trauma, consisting of a 3.0-cm incision, probe manipulating for 5 min and finger manipulating 10-cm intestine for 5 min with 0.5 s for a time period, both with a doubled frequency. To limit variability, all surgeries were performed by the same person and surgery proceeding time was monitored. Tibial surgery and abdominal surgery were completed within 10−15 min, and extended abdominal surgery was completed within 25−30 min. While no procedures were performed in the control group.

Anesthesia was achieved using a sealed chamber to deliver 3−5% sevoflurane with 100% oxygen and then maintained with 2−3% sevoflurane-O_2_ by mice anesthesia mask connected to an animal anesthesia device (R500SE, RWD Life Science, Shenzhen China). Anesthesia depth was monitored and adjusted according to body movement and respiratory rate. The incision was infiltrated with ropivacaine (1.0% and 0.1 ml) and dressed with polysporin to prevent potential infection after surgery.

### Behavioral tests

**FIGURE 1:**
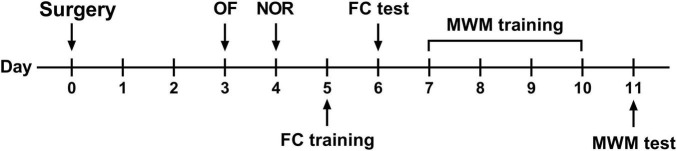
Behavioral experimental flow. OF, open field test; NOR, novel object recognition test; FC, fear conditioning; MWM, Morris water maze.

All mice were kept undisturbed on postoperative days 1 and 2 to enhance recovery. Behavioral tests were initiated on postoperative day 3, as described in our previous study (Meng et al., 2019). All behavioral tests were conducted in a room adjacent to the housing room with dim light. The behavioral experiment design is shown in Figure 1.

#### Open field

The open field test was performed on postoperative day 3 to assess the motor activity, exploratory activity, and anxiety-like behavior in mice. A square (40 × 40 × 40 cm) box was divided into one central area and several peripheral areas. The mouse was placed in the central area and allowed to freely move about with a camera recording for 5 min and footage analyzed using a computerized image analyzing system (ImageMD3000, ZhengHua Biologic Apparatus, Huaibei, China).

#### Novel object recognition

The novel object recognition test was performed on postoperative day 4 to assess visual and short-term spatial memory using the same apparatus for OF. After habituation, the mice facing the wall were placed into the box with two identical objects and allowed to explore for 5 min. After 1 h, one object was replaced with a novel object, and the mice was on test for 5 min. Unlike typical NOR, to minimize the effect of impaired mobilization by surgery, here, we recorded and analyzed both the amount of time and distance taken to explore each object. The recognition index (RI) = exploring time or distance for novel object/total exploring time for objects.

#### Fear conditioning

The fear conditioning test is used to assess fear memory. In a conditioning chamber (30 × 30 × 45 cm), the moving paths and freezing behavior of the animals were recorded and analyzed (SuperFcs, XinRuan Information, Shanghai, China). On postoperative day 5, after 180 s of free exploring in the chamber, the mice were then presented with an auditory cue (70 dB, 3 kHz, conditional stimulus) for 30 s, immediately followed by an unconditional stimulus, a 2-s foot-shock (0.75 mA). This procedure was repeated with an interval of 60 s, and the mice were then removed from the chamber 30 s later. On postoperative day 6, the mice were put in the same chamber, with no tones or foot-shocks delivered, and the percent of freezing was recorded to assess contextual fear memory, which indicates hippocampus-dependent memory.

#### Morris water maze

The Morris water maze was used to assess spatial learning and memory. A water-filled circular tank was surrounded by visual cues and divided into four quadrants, with a platform below the water surface in the target quadrant. Acquisition started on postoperative day 7, and a probe trial test was conducted on postoperative day 11. In the acquisition session, a trial was terminated when the mice reached the platform, where it was allowed to stay for 15 s. If the animal failed to find the platform within 60 s, it was manually guided to the platform and left there for 15 s. During the probe trial, the platform was removed, and the search pattern of the mice was tracked again. The moving paths and time required for locating the hidden escape platform or the target quadrant were analyzed with a video tracking system (EthoVision XT, Noldus Instruments, Wageningen, Holland). Similar to NOR, both the time and distance spent in the target quadrant were analyzed for spatial memory.

### Behavioral Z-score calculation

To obtain a comprehensive and integrated memory performance profile, the Z-score methodology was adopted (Meng et al., 2019). For mean normalization of results, the Z-score for each individual was calculated using the following formula:


$$Z=\frac{X-\mu }{\sigma }$$

X represents individual data for the observed parameter. μ and σ represent the mean and the standard deviation of the control group, respectively. The Z-score indicates how much standard deviation (σ or SD) an observation (X) is above or below the mean of a control group (μ).

Meanwhile, we need to address a couple of issues about the Z-score here. First, according to the formula, a higher positive Z-score indicates poorer performance. Second, to avoid any weighted effect of locomotion, a designated Z-score was obtained in NOR and MWM for each animal by averaging the Z-scores of time-related and distance-related parameters. Based on a bold hypothesis that the behavioral dimensions of NOR, FC, and MWM were of equal weight, an overall hippocampus-dependent memory Z-score was obtained for each animal based on the three tests:


$$H\,i\,p\,p\,o\,c\,a\,m\,p\,a\,l\,M\,e\,m\,o\,r\,y\,S\,c\,o\,r\,e=\frac{Z_{N\,O\,R}+Z_{F\,C}+Z_{M\,W\,M}}{3}$$

### Enzyme-linked immunosorbent assays

All mice were decapitated under anesthesia with sevoflurane, and the brains were quickly removed and dissected to collect the hippocampus. The samples were rinsed with cold saline and homogenized for ELISA of TNF-α, IL-1β, and IL-6. The absorbance was read at 450 nm using a microplate spectrophotometer (Thermo Fisher Scientific Inc., United States). The concentrations were calculated according to the standard curve, and values were presented as picogram per milligram. The protocol, reagent preparation, and working standards all complied with the manufacturer’s instructions (R&D Systems, United States).

### Quantitative real-time polymerase chain reaction

Total RNA was extracted from the homogenized hippocampus using the TRIzol Reagent protocol (Life USA). cDNA was generated by using a HiFiScript cDNA Synthesis Kit (ComWin Biotech, Beijing, China). Next, target cDNA (TNF-α, IL-1β, IL-6, and HMGB-1) and reference cDNA (β-actin) were amplified simultaneously by qPCR using an UltraSYBR Mixture Kit (ComWin Biotech, Beijing, China). The amplification temperature should follow the following protocol: 95°C for 10 min, followed by 40 cycles of 95°C for 15 s and 60°C for 30 s. Data were analyzed using the comparative threshold cycle (Ct) method, and all values were expressed relative to the expression of β-actin (2^–ΔΔ*ct*^).

### Immunohistochemistry

After anesthesia, the mice were perfused transcardially with saline, followed by 4% paraformaldehyde. The brains were dissected out, fixed in 4% PFA overnight, and then transferred to sucrose solution (15% for 24 h and then 30% for 24 h). The brains were frozen in OCT compound (Sakura Finetek, CA, United States) and cut into 25-μm-thick sections (CM1950, Leica, Frankfurt, Germany). The sections containing the hippocampus were incubated at 4°C overnight in 0.1 M PBS buffer containing 0.5% TritonX-100 and the primary antibodies goat anti-Iba1 (1:500, Abcam, Cambridge, United States). The sections were then washed three times (8−10 min) with PBS solution and incubated for 90 min at room temperature in the same buffer containing Alexa 488-conjugated secondary antibodies donkey anti-goat (1:500, Abcam, Cambridge, United States). Images were obtained under a confocal laser scanning microscope (SP8, Leica, Frankfurt, Germany). A total of three sections per mouse were imaged. Quantification of Iba-1 staining was analyzed by ImageJ analysis software (NIH, United States) and performed in a blinded fashion.

### Statistical analysis

All data are expressed as mean ± standard error of the mean (SEM) unless stated otherwise. Prism 8 (GraphPad Software, San Diego, CA, United States) was used for one-way and two-way analyses of variance (ANOVAs), followed by Bonferroni’s *post hoc* tests. IBM SPSS Statistics 20 (IBM Corp, Zurich, Switzerland) was used for Student’s *t*-test, analysis of covariance (ANCOVA), Pearson’s correlation, and chi-squared test. The analyzed statistic difference is indicated in figure legends. *P* < 0.05 was considered statistically significant.

## Results

### Mortality, body weight loss, and locomotion changes after surgery

**FIGURE 2:**
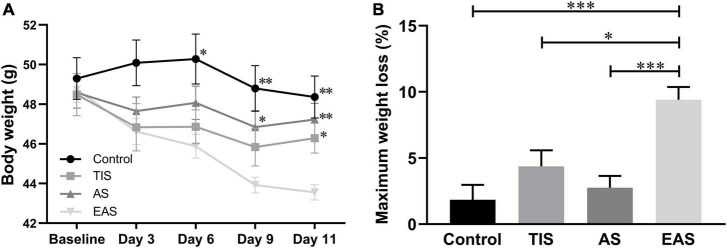
Body weight changes after surgery. **(A)** There was a significant weight loss in mice that underwent extended abdominal surgery on postoperative days 6, 9, and 11. *, ^**^, ^***^ vs. group EAS. **(B)** On day 11, after all behavior tests were completed, group Ex-Abdo mice lost significantly more weight than the other three groups of mice. *, ^***^ vs. group EAS. Data represent mean ± SEM (*n* = 9–12 per group). *P < 0.05; ^**^P < 0.01; ^***^P < 0.001.

During the behavioral testing procedure, three mice (25%) that underwent EAS died, one on postoperative day 1 and two during MWM training. Also, one of the mice that underwent TIS was excluded due to sciatic nerve injury. No mice died in the other two groups. A significant weight loss trend was detected in mice undergoing EAS in the behavioral experiments (Figure 2A). At the end of multiple behavioral tests, the maximum weight loss of the EAS group was significantly higher than that in the other three groups (Figure 2B).

**FIGURE 3:**
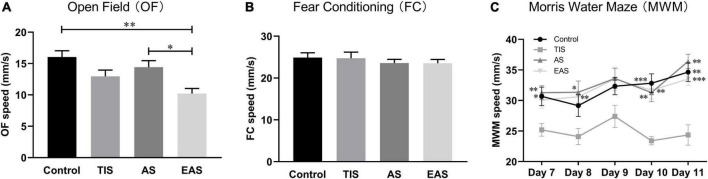
Locomotion among behavioral tests. **(A)** In OF, the average speed of mice exploring after extended abdominal surgery was significantly lower than that in controls and abdominal surgical mice. **(B)** In the FC, there were no significant differences between groups. *, ^**^ vs. group EAS. **(C)** In the MWM, the swimming speed of tibial fracture mice was significantly lower than that of other groups. *, ^**^, ^***^ vs. group TIS. Data represent mean ± SEM (*n* = 9–12 per group). *P < 0.05; ^**^P < 0.01; ^***^P < 0.001.

Regarding locomotion, the average moving speed was recorded to investigate the trends across tests. In the OF test, EAS mice had a significant deterioration in locomotion performance compared with control and AS mice (Figure 3A). During the FC training phase, no difference was found in locomotion between groups (Figure 3B). In the MWM, TIS mice had a significant swimming ability deterioration compared with other mice groups (Figure 3C).

### Emotional behavior changes after surgery

**FIGURE 4:**
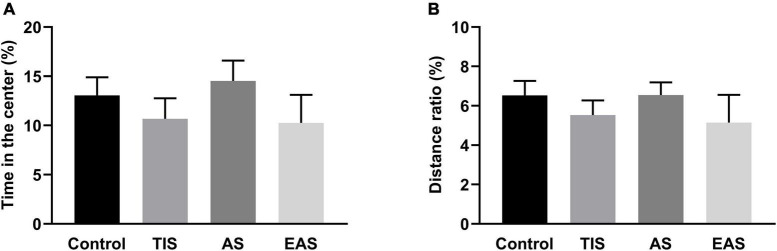
Time in the center **(A)** and distance in the periphery/total distance ratio **(B)** of OF. There was no significant difference in time in the center and distance in the periphery/total distance ratio between groups. Data represent mean ± SEM (*n* = 9–12 per group).

The OF test was performed on day 3, and each group had no difference in the time in the center (Figure 4A), consistent with no significant differences found in the periphery/total distance ratio (Figure 4B). Therefore, the anxiety- and depressive-like behaviors were not influenced by surgical trauma types.

### Hippocampus-dependent memory changes after surgery

**FIGURE 5:**
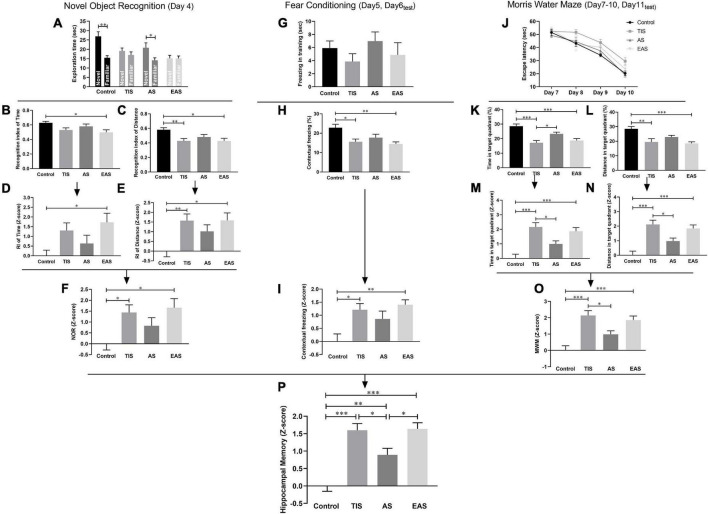
Learning and memory behavior and Z-scores in the NOR, FC, and MWM. **(A)** Time mice spent exploring novel and familiar objects in the NOR test. **(B)** RI based on time. **(C)** RI based on distance. **(D,E)** Z-score normalization was performed with the same statistical results as before normalization. **(F)** NOR Z-scores were obtained by averaging the two index Z-scores. **(G)** Freezing time in the training phase of the FC test. **(H)** Contextual freezing time. **(I)** Normalization of data using the Z-score method was performed. **(J)** Escape latency in the MWM test. **(K,L)** Time and distance in the target quadrant. **(M,N)** Z-score normalization was performed with similar differences between groups as before normalization. **(O)** MWM Z-scores were obtained by averaging the two ratio Z-scores. **(P)** Hippocampus-dependent memory Z-score calculated by averaging the Z-scores of NOR, FC, and MWM. One-way ANOVA followed Bonferroni’s *post hoc* test. Data represent mean ± SEM (*n* = 9–12 per group). *P < 0.05; ^**^P < 0.01; ^***^P < 0.001.

The NOR results showed that both control and AS mice were able to discriminate between familiar and novel objects, while the TIS and EAS mice were not (Figure 5A). In the RI based on time, EAS mice had poorer performance than controls (Figure 5B). Meanwhile, upon distance-based RI, both EAS and TIS mice had poorer performance than controls (Figure 5C). Based on these data, Z-score transformation was performed within the two NOR parameters, yielding the same *p*-Values as before normalization (Figures 5D,E). Then, the average of two normalized Z-score values generated a single value per mouse: the NOR Z-score, which comprehensively reflected the short-term visual recognition memory. TIS and EAS mice showed more impairment in memory than controls (Figure 5F).

In the FC test, after a day of training in the contextual fear test, a decreased freezing time was observed in TIS and EAS mice (Figure 5H). The Z-score transformation was performed within contextual freezing time, resulting in the same *p*-values as before normalization (Figures 5G,I).

In the MWM, after the acquisition phase, all groups were able to learn the task, as indicated by the decreased latency to find the hidden platform across trials (Figure 5J). Considering the significant differences in swimming speed between groups (Figure 3C), ANCOVA was used to examine the effects of surgical trauma on latency using speed as the covariate, and no significant difference between the groups was detected. Regarding locomotion differences between the groups, time and distance percentages in the target quadrant were combined to analyze the performance in the probe trial. Compared to controls, TIS and EAS mice not only spent more time (Figure 5K) but swam longer distances in the target quadrant (Figure 5L). Similarly, the Z-scores of time and distance in the target quadrant were calculated (Figures 5M,N) and averaged to obtain the MWM Z-score (Figure 5O), and we found that spatial memories were significantly disrupted by TIS and EAS.

Based on the hypothesis that all the three tests are similarly weighted, integrated behavioral Z-scoring was employed to investigate the potential of combining results across NOR, FC contextual fear test, and MWM for hippocampus-dependent learning and memory-related behavior. The integrated Z-score analysis revealed that hippocampus-dependent memories were significantly impaired after each type of surgery (Figure 5O). Moreover, a higher impairment was detected after TIS and EAS than after AS.

### Hippocampal inflammation after behavioral tests

**FIGURE 6:**
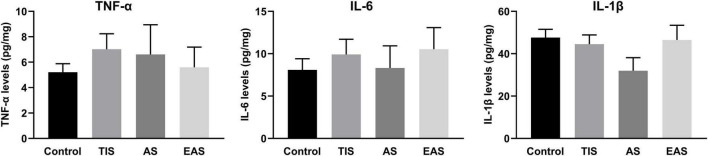
Hippocampal inflammatory cytokine levels after the end of all behavioral tests. There were no significant differences between groups in TNF-α, IL-6, and IL-1β levels in the hippocampus. Data represent mean ± SEM (*n* = 9–12 per group).

After completing all behavioral tests on day 12, all mice of the four groups were killed, and their hippocampus harvested for ELISA of TNF-α, IL-6, and IL-1β cytokines. The results showed that inflammatory cytokines had no significant differences between the groups (Figure 6), indicating that hippocampal inflammation was not significantly affected by either surgery type on day 12.

### Hippocampal inflammation on postoperative days 1, 3, and 7

**FIGURE 7:**
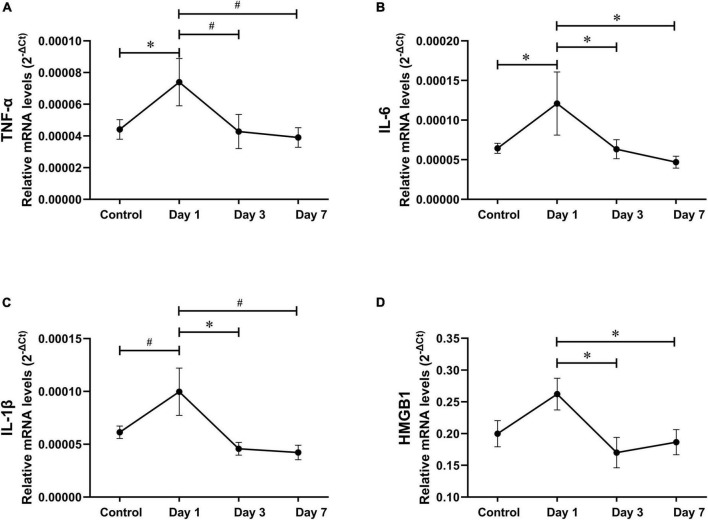
Hippocampal inflammatory trend after extended abdominal surgery. **(A–D)** TNF-α, IL-6, IL-1β, and HMGB-1 mRNA levels on postoperative days 1, 3, and 7. Data represent mean ± SEM (*n* = 9–12 per group). ^#^*P* < 0.01; *P < 0.05.

It has been reported that hippocampal pro-inflammatory factors significantly increased at 24 h, rather than 7 days after abdominal surgery (Zhang Z. et al., 2016), similar to our findings regarding tibial fracture surgery (Meng et al., 2019). To compare neuroinflammation between the three surgery types, we analyzed the postoperative hippocampal inflammatory trend in EAS. Compared to the controls, hippocampal TNF-α and IL-6 mRNA levels significantly increased on the first day after EAS but almost decreased to control levels on days 3 and 7 (Figure 7).

### Hippocampal pro-inflammatory cytokine expressions on postoperative day 1

**FIGURE 8:**
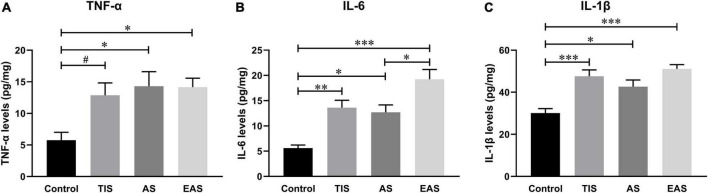
Hippocampal inflammatory cytokine levels on postoperative day 1. **(A)** Compared with controls, TNF-α levels significantly increased in abdominal and extended abdominal surgical mice on postoperative day 1. **(B)** IL-6 levels significantly increased on postoperative day 1 after each type of surgery, with a higher level after extended abdominal surgery than that after abdominal surgical mice. **(C)** IL-1β levels were also significantly increased after all three types of surgery, however, with no significant differences among groups. Data represent mean ± SEM (*n* = 6). ^#^*P* < 0.1; *P < 0.05; ^**^P < 0.01; ^***^P < 0.001.

The hippocampal inflammatory peak seemed to appear on postoperative day 1 in all three surgery types. Considering previous studies used postoperative day 1 as the time point to investigate postoperative neuroinflammation (Terrando et al., 2011; Zhang et al., 2014; Zhang Z. et al., 2016), we also compared the effects of the three surgery types on hippocampal inflammation on postoperative day 1. Compared to the controls, all three inflammatory cytokines significantly increased on day 1 after each type of surgery. However, cytokine levels had no significant differences between the surgical groups (Figure 8).

### Hippocampal microglial activation on postoperative day 1

**FIGURE 9:**
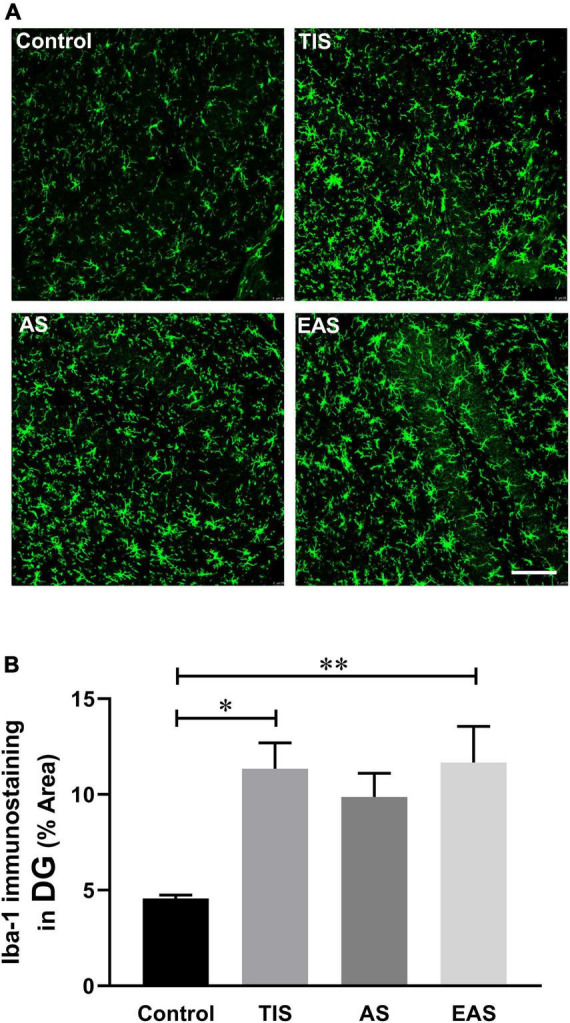
Hippocampal microglial activation on postoperative day 1. **(A)** Representative image staining in the hippocampal DG region. Scale bar: 50 μm. **(B)** Microglia in the hippocampal dentate gyrus region were significantly activated after TIS and EAS. No significant differences were found between the three surgical groups. Data represent mean ± SEM (*n* = 6). *P < 0.05; ^**^P < 0.01.

Apart from pro-inflammatory cytokine levels, glial cell activation, microglia in particular, could also be a hippocampal neuroinflammation indicator (Wang et al., 2020; Saxena et al., 2021). Therefore, we examined the expression of the activated microglial marker Iba-1 on postoperative day 1. The microglia in the hippocampal dentate gyrus (DG) region were significantly activated after TIS and EAS; however, no significant differences were found between the three surgical groups (Figure 9).

## Discussion

The major findings of this study are summarized in Table 1. Different types of non-cardiac surgical trauma can have different effects on locomotor activity. Compared with AS, TIS and EAS led to more significant cognitive impairment. On postoperative day 12, with all behavior tests completed, the levels of hippocampal inflammatory cytokines in all three surgery groups dropped to the level of the control. Inflammatory cytokines peaked on postoperative day 1 and decreased to control levels on days 3 and 7. Hippocampal neuroinflammation showed no significant difference between the three surgery types on postoperative day 1.

**TABLE 1:** Effects of three surgical trauma types in body weight loss, locomotor activity, emotional behavior, cognitive behavior, and neuroinflammation.

Body weight loss	Locomotor activity		Emotional behavior	Cognitive behavior	Neuroinflammation (postoperative day 1)
	Motor ability	Swimming ability			
EAS > AS = TIS	AS = TIS > EAS	AS = EAS > TIS	AS = TIS = EAS	TIS = EAS > AS	AS = TIS = EAS

### Initial impression of the three surgical trauma

In this study, AS was performed following the standard protocol in time lasted and manipulating frequency, while EAS was prolonged to 10 min with a doubled manipulating frequency. EAS was indeed more traumatic than the other two surgery types due to a higher postoperative mortality rate; 11 out of 30 mice died in previous pilot experiments while no death was found in either TIS or AS group, and EAS mice showed a more significant decrease in weight than in the other groups.

Based on the purpose and hypothesis of the study, EAS was supposed to be the experimental group with higher trauma than “abdominal surgery.” Tissue oxygenation and perfusion were monitored with the respiratory rate maintained at 92−120 bpm, and the lip color was closely observed during surgery. Aseptic techniques were employed during the surgery, but with no infection indicators measured, sepsis could not be ruled out. This is a limitation of this study, but on the other hand, the mice undergoing major abdominal surgery might have a higher death rate than observed in real-world practice.

Locomotor activity is also related to behavioral outcomes. Therefore, we compared the effects of three surgery types on locomotor activity. Only the EAS mice were found with declined ground locomotion activity in the OF test, consistent with the previous finding that locomotor activity in the OF was not affected by TIS (Feng et al., 2013; Sun et al., 2017). While the MWM data indicated TIS mice had decreased swimming ability, different from the previous evidence (Feng et al., 2013; Jiang et al., 2019; Zhan et al., 2019). Accordingly, considering that the locomotor activity of the mice was significantly affected during behavioral tests, the Z-normalization methodology was introduced to reduce statistical bias.

### Cognitive changes after surgeries

Unlike dementia syndrome in Alzheimer’s disease, cognitive impairment in patients after surgery may not be perceptible. This usually hinders POCD diagnosis because those asymptomatic patients may not receive neuropsychological tests, which is crucial for diagnosis (Evered et al., 2018). Hence, many POCD preclinical studies employed certain behavioral test batteries to investigate cognitive changes in animals, and similar to clinal practice, the hippocampus-dependent memory was the focus of attention (Zhang et al., 2014; Huang et al., 2018; Yao et al., 2019). Among animal studies, OF, NOR, FC, and MWM tests are frequently used to assess cognitive changes, especially for hippocampus-dependent memory. Therefore, these tests were also chosen in the present study and arranged in an order of increasing stress intensity.

In the OF test, we found that emotional behaviors were not affected after each surgery type as demonstrated by other studies (Feng et al., 2013; Jia et al., 2015; Xiao et al., 2018). However, Hovens et al., 2013, 2014, 2015a,^2016^ and other studies (Lu et al., 2015; Terrando et al., 2016) revealed that surgical trauma could affect anxiety- and depressive-like behaviors in animals. These contradictory results might be a result of different surgical trauma types applied and/or the different ages of animals since aging plays a vital role in the development of dementia and depression. In the FC test, TIS and EAS mice demonstrated a significant reduction in freezing behavior in contextual-dependent fear tests, which mainly reflect the hippocampus-depende

nt memory. These results are in agreement with the findings of Terrando et al. (2010, 2011, 2015) and many other researchers (Zhang et al., 2014; Qiu et al., 2016; Sun et al., 2017; Xiao et al., 2018; Chen et al., 2019). Meanwhile, these studies demonstrated that the cue-dependent freezing behavior was not affected after surgery. In the MWM test, we did not find significant differences in latency across training phases between the groups. Combined with FC results, our current findings indicated that surgical trauma does not affect the learning process as proved by other studies (Wang Y. et al., 2016; Ma et al., 2017).

Cellular damage after surgical trauma triggers endogenous factors known as damage-associated molecular patterns (DAMPs), which activate immune cells such as neutrophils and monocytes to resolve the damage and restore homeostasis (Yang et al., 2020). Activation of these cells contributes to systemic inflammation, which can impact the brain. It should be emphasized that injury is a key driver of inflammation, and different intensities of surgical trauma ultimately lead to different degrees and types of cognitive impairment, just as the behavioral differences caused by the three surgeries in this study.

In addition to surgical trauma, the differences between studies can be attributed in part to the animal’s age. In POCD animal studies, the eldest animals were 24−25 months (Barrientos et al., 2012), and the youngest were 6−10 weeks (Lu et al., 2015). Many studies compared the effects of surgery on adult and aging animals, with the results consistently suggesting that adult animal cognitive behavior was not affected, while older animals were affected (Li et al., 2014; Kawano et al., 2015; Xu et al., 2019). In this study, ICR mice of 12−14 months were used to avoid failure on behavioral tests, due to age-related incompetency. Meanwhile, other studies used mice of the same age (Gambus et al., 2015; Li et al., 2016; Wang et al., 2017).

### Application of behavioral Z-scores

As a commonly applied method in clinical trials, Z-scores consider differences from mean group values regarding SDs from the control or baseline means. Here, we employed Z-scores also because the locomotion in several behavioral tests was found impaired by surgical trauma. Different preclinical studies have used Z-scores to analyze animals’ emotionality by combining different behavioral tests into a single apparatus (Guilloux et al., 2011; Tian et al., 2015; Labots et al., 2018; Meng et al., 2019). Peng et al. (2016) attempted to use Z-scores to study postoperative delirium in mice. In addition to integrated Z-scores, based on the SD of Z-normalization, values that are similar across parameters and tests were also introduced. Averaging *Z*-values should avoid the weighted effects of one parameter or one test over another. Our previous study presents a particular and comprehensive explanation of this topic (Meng et al., 2019). Moreover, considering the results from the hippocampus-dependent memory tests, the integrated behavioral Z-score could amplify and quantify the effect of interventions, represented here by surgical trauma.

**FIGURE 10:**
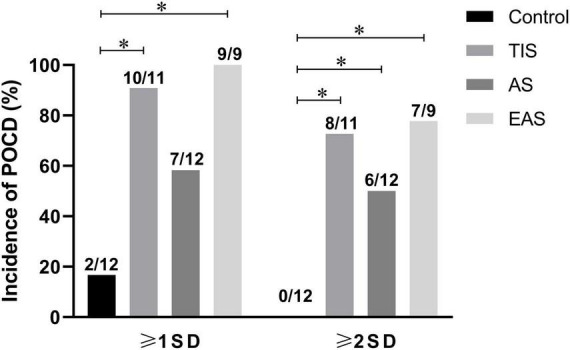
Incidence of POCD as defined by SD criterion. According to the 1-SD criterion, the incidences of POCD in TIS and EAS mice were higher than those in controls. According to the 2-SD criterion, the incidences of major POCD in all three types of surgical mice were higher than those in controls. The chi-squared test was used for most data analysis, and the significance level was adjusted to 0.0083 according to Bonferroni’s method. *P < 0.0083.

Based on the original purpose of this study, the Z-normalization method might reduce the gap between POCD clinical and preclinical perspectives. According to an international study of postoperative cognitive dysfunction (ISPOCD 1), POCD was only present in 25.8% at 1 week and 9.9% at 3 months after major non-cardiac surgery in patients younger than 60 years (Moller et al., 1998). However, in almost all POCD animal studies, surgical group animals were classified as POCD, while no diagnostic criteria for POCD were established. Therefore, some of these negative results might be false negatives. Zhan et al. (2019) categorized mice into POCD and non-POCD groups using hierarchical cluster analysis. Few researchers rediagnosed and further reclassified surgery and anesthesia groups. In the current study, Z-normalization within and across different behavioral tests resulted in a single score per mouse, which might be seen as a quantitative “diagnosis” of their hippocampus-dependent memory. According to “Recommendation 2018”^4^, another clinical analysis approach, the SD criterion, was tentatively used in the current animal study. The SDs were calculated from the integrated Z-scores of controls. Hence, mild POCD was diagnosed as the individual integrated Z-score increased ≥ 1-SD, while major POCD was defined as an increased integrated Z-score ≥ 2-SD^4^ (Figure 10). The results showed that almost all mice developed POCD after tibial TIS and EAS, significantly different from the clinical setting. The surgical trauma used in POCD preclinical animal models is too large to imitate the clinical practice, which might explain the differences discussed earlier.

### Hippocampal neuroinflammation after surgery

The central inflammation hypothesis of POCD can be summarized as that surgery on peripheral tissues and organs induces systemic inflammation that can be transferred into the brain, especially to the hippocampus, and induce neuroinflammation (Yang et al., 2020; Saxena et al., 2021). Regarding postoperative changes of systemic inflammation, studies on different surgery types have shown that the inflammation peaked between 3 and 6 h, returning to the baseline at 24 h (Hovens et al., 2013, 2014, 2015b; Yang et al., 2017). However, compared to systemic inflammation, the central inflammation tendency after surgery is controversial. To compare the neuroinflammation between different surgical trauma types, we preliminarily proved that hippocampal neuroinflammation levels peaked on postoperative day 1 and decreased almost to control levels on days 3 and 7 in tibial and abdominal surgeries (Meng et al., 2019). Furthermore, we found that hippocampal inflammatory cytokines were not significantly affected by surgery types on day 12 after all behavioral tests were completed. Unfortunately, hippocampal inflammatory cytokines were not studied within the first 24 h after surgery. It has been reported that pro-inflammatory cytokines started to increase upon 2 h postoperatively, peaked at 6 h, and then dropped to normal levels, similar to the trend of systemic inflammation (Zhao et al., 2016; Xiang et al., 2019). However, it has also been shown that hippocampal TNF-α, IL-6, and IL-1β in surgical animals present a clear increase in days 3 (Zhang X. et al., 2016), 4 (Barrientos et al., 2012), and 7 (Qiu et al., 2016), and even at 2 (Hovens et al., 2016), 3 (Hovens et al., 2014), and 4 weeks (Wang H. L. et al., 2016).

Moreover, we analyzed microglial activation as another neuroinflammation indicator. However, the trend of hippocampus microglial activation was not repeated. It has been suggested by some researchers that the duration of postoperative microglial activation is consistent with that of increased pro-inflammatory cytokine levels. Most of them reported that microglial activation had returned to normal levels on the postoperative day 7 (Cibelli et al., 2010; Zhang Z. et al., 2016; Duan et al., 2018; Yang et al., 2019). However, compared to increased pro-inflammatory cytokine levels, microglial activation can last for a longer time after surgery, showing a certain delay (Hovens et al., 2013; Zhao et al., 2016; Duan et al., 2018; Lu et al., 2019). Furthermore, these differences might be attributed to different surgical trauma types.

Since the levels of the pro-inflammatory cytokines peaked on day 1, we analyzed the level of hippocampal inflammation among surgery types on day 1, which was also adopted by many research teams as the single observation point (Zhang et al., 2014; Zhao et al., 2016; Vacas et al., 2017; Liang et al., 2018; Ni et al., 2018; Xiong et al., 2018; Lu et al., 2019). No significant differences in microglial activation and inflammatory levels in the hippocampus were detected between the three surgical trauma types, opposing the behavior performance resulting from TIS and EAS in greater cognitive impairment compared to AS. This conflict may be contributed to the small sample size or the variability of the experimental animals. However, we cannot completely deny this contradiction. Previously, some researchers analyzed the association between behavioral tests and hippocampal neuroinflammation. For example, Hovens et al. (2014) found that exploratory behavior, but not learning and memory, was correlated with plasma IL-6 levels at postoperative 24 h. Also, CA1 microglial activation was correlated with the performance of, FC, and Y-maze on postoperative day 43 (Hovens et al., 2013). Another study reported an inverse relationship between hippocampal TNF-α and IL-1β levels, and NOR performance on postoperative day 7 (Kawano et al., 2015; Locatelli et al., 2018). However, in the present study, no correlation was found, which might be attributed to the fact that pro-inflammatory cytokines have dropped to baseline when the behavior tests ended.

## Conclusion

Overall, the three types of non-cardiac surgical trauma manifested differently regarding mortality, body weight loss, and locomotion changes. To a certain extent, these differences might give rise to the different degrees of hippocampus-dependent memory impairment, which were comprehensively and quantitatively demonstrated using the Z-score methodology. However, no significant differences in hippocampal neuroinflammation were detected. This contradiction might be explained by experimental errors, animal variability, or small sample size. Moreover, the gap between POCD clinical and preclinical studies should be cautiously considered. The results from the SD method tentatively used in the present animal experiments could partly interpret this gap. Furthermore, including the present study, we have been investigating the poor relationship between neuroinflammation and cognitive impairment after surgery (Meng et al., 2019). It should be clarified that the neuroinflammation hypothesis of POCD originates from and accumulates its evidence mainly in preclinical studies, but not clinical trials. For example, a small sample size trial investigated 10 patients and found no correlation between postoperative cognitive impairment and cytokines (Hirsch et al., 2016). Several randomized controlled trials showed that perioperative application of hormones neither reduce POCD risks (Ottens et al., 2014) nor attenuate neuroinflammation (Danielson et al., 2018). Considering the inconsistency between clinical trial and preclinical study results, a consensus on the POCD preclinical study should be constructed, similar to the “Recommendation 2018,” to reduce the gap between clinical and preclinical studies by standardizing animal models, methodologies, and research directions.

## Data availability statement

The raw data supporting the conclusions of this article will be made available by the authors, without undue reservation.

## Ethics statement

The animal study was reviewed and approved by the Animal Care and Use Committee of Ningbo University.

## Author contributions

BL, BM, and JC contributed to the conception and design of the study. BL wrote the manuscript. XZ organized the database. LM, DS, RL, HZ, and RW conducted the study and included the data collection and analysis. All authors participated in manuscript revision, proofreading and approved the submitted version.

## Abbreviations

POCD: postoperative cognitive dysfunction
TIS: tibial surgery
AS: abdominal surgery
EAS: extended abdominal surgery
OF: open field
NOR: novel object recognition
MWM: Morris water maze
RI: recognition index
ELISA: enzyme-linked immunosorbent assays
qRT-PCR: quantitative real-time polymerase chain reaction
ANOVA: analysis of variance
DG: dentate gyrus
DAMPs: damage-associated molecular patterns
SD: standard deviation
SEM: standard error of the mean
